# Immunoinformatics approach for predicting epitopes in HN and F proteins of *Porcine rubulavirus*

**DOI:** 10.1371/journal.pone.0239785

**Published:** 2020-09-25

**Authors:** Luis I. Siañez-Estrada, José F. Rivera-Benítez, Nora H. Rosas-Murrieta, Julio Reyes-Leyva, Gerardo Santos-López, Irma Herrera-Camacho

**Affiliations:** 1 Laboratorio de Bioquímica y Biología Molecular, Centro de Química, Instituto de Ciencias, Benemérita Universidad Autónoma de Puebla, Puebla, México; 2 Posgrado en Ciencias Químicas, Benemérita Universidad Autónoma de Puebla, Puebla, México; 3 Laboratorio de Biología Molecular y Virología, Centro de Investigación Biomédica de Oriente, Instituto Mexicano del Seguro Social (IMSS), Metepec, México; 4 Centro Nacional de Investigación Disciplinaria en Salud Animal e Inocuidad, Instituto Nacional de Investigaciones Forestales, Ciudad de México, México; Macfarlane Burnet Institute for Medical Research and Public Health, Australia, AUSTRALIA

## Abstract

*Porcine rubulavirus* (PRV), which belongs to the family *Paramyxoviridae*, causes blue eye disease in pigs, characterized by encephalitis and reproductive failure in newborn and adult pigs, respectively. There is no effective treatment against PRV and no information on the effectiveness of the available vaccines. Continuous outbreaks have occurred in Mexico since the early 1980s, which have caused serious economic losses to pig producers. Vaccination can be used to control this disease. Searching for effective antigen candidates against PRV, we first sequenced the PAC1 F protein, then we used various immunoinformatics tools to predict antigenic determinants of B-cells and T-cells against the two glycoproteins of the virus (HN and F proteins). Finally, we used AutoDock Vina to determine the binding energies. We obtained the F gene sequence of a PRV strain collected in the early 1990s in Mexico and compared its amino acid profile with previous and more recent strains, obtaining an identity similarity of 97.78 to 99.26%. For the F proteins, seven linear B-cell epitopes, six conformational B-cell epitopes and twenty-nine T-cell MHC class I epitopes were predicted. For the HN proteins, sixteen linear B-cell epitopes, seven conformational B-cell epitopes and thirty-four T-cell MHC class I epitopes were predicted. The ATRSETDYY and AAYTTTTCF epitopes of the HN protein might be important for neutralizing the viral infection. We determined the *in silico* binding energy between the predicted epitopes on the F and HN proteins and swine MHC-I molecules. The binding energy of these epitopes ranged from -5.8 to -7.8 kcal/mol. The present study aimed to assess the use of HN and F proteins as antigens, either as recombinant proteins or as a series of peptides that could activate different responses of the immune system. This may help identify relevant immunogens, saving time and costs in the development of new vaccines or diagnostic tools.

## Introduction

*Porcine rubulavirus* (PRV), also known as La Piedad Michoacán virus (LPMV), is a member of the *Paramyxoviridae* family. PRV causes the blue eye disease (BED) in pigs. BED is characterized by uni- or bilateral corneal opacity, respiratory distress and progressive neurological signs, with high rates of mortality in piglets and reproductive failure in adults [1–3].

PRV was first isolated in La Piedad, Michoacan, Mexico, in 1980. There have been several outbreak reports (1984–2015). In 1997, PRV was isolated from different outbreaks; these isolates were named Produccion Animal Cerdos (PAC). In 2015, an outbreak of BED was observed in Central Mexico; it was characterized by an increase in the number of adult pigs with neurological signs. The seroprevalence of PRV ranged from 9 to 23.7%, and there were PRV reports in 16 states of Mexico. The most affected states were Guanajuato, Jalisco and Michoacan [3–5]. The diagnosis of BED is usually stablished by RT-PCR, although ELISA may be used too. There is no specific treatment against PRV but there are two PRV vaccines commercially available, both of which are based on inactivated viruses; however, there are no studies showing their level of protection against PRV.

PRV is an envelope virus with a structural organization similar to that of other rubulaviruses such as the mumps virus (MuV) and the parainfluenza virus 5 (PIV5). The viral particle is composed of six structural proteins: the nucleoprotein (NP), the large protein (L), the phosphoprotein (P), the matrix protein (M) and two surface glycoproteins, hemagglutinin-neuraminidase (HN) and the fusion (F) protein [6]. HN recognizes and binds to a receptor containing sialic acid (sialyl (α2,3) lactose) on the host cell, while the F protein is a fusion protein that promotes the fusion of cell and viral membranes, facilitating the penetration of virion material into the cell. The F protein is synthesized as a precursor (F0) that is proteolytically processed, resulting in the disulfide-linked F2 and F1 polypeptides. Substitutions of cleavage sites in the amino acid sequence has been associated with virulence in the Newcastle disease virus. The N-terminus of F1 contains the active fusion peptide that participates in the fusion between the viral and the host cell membranes [7–10].

As with other paramyxoviruses, HN is the immunodominant protein in PRV. During PRV infection, the pig’s immune system produces antibodies capable of recognizing HN, M and NP proteins. In experimental infected pigs, the production of antibodies against the HN protein of PRV starts at week two post-infection [11].

Due to its complex properties and the fact that it is a surface protein, the host’s immune response exerts selection pressure on HN. This makes aminoacidic changes and antigenic variations a common occurrence; these mutations are important because they make it impossible to use only one antigen to protect against all PRV variants [12, 13].

The expression of two variants of recombinant HN capable of inducing antibodies in mice has been reported as part of the effort to look for an effective vaccine against PRV [14, 15]. Indeed, our research group has demonstrated that murine antibodies generated against recombinant HN proteins can neutralize PRV infection in cell cultures [14].

The production of antibodies against the F protein has been demonstrated in infections with other paramyxoviruses [16, 17]. It has been proposed to use the F protein as a vaccine antigen against PIV5 and MuV, since specific antibodies can neutralize the infectious activity of these viruses and reduce syncytia-mediated viral spread [18, 19]. Moreover, *in silico* analysis has demonstrated that some antigenic determinants are conserved in the MuV F protein [20].

Developing effective vaccines against viral diseases is an urgent matter, and PRV infection is no exception, especially given the genetic variations observed in recent studies, which cast a doubt on the effectiveness of existing vaccines [4, 5, 21].

The present study determined the F gene sequence of a PRV isolate from the early 1990s and compared this sequence with that of other isolates. We analyzed the F and HN sequences in order to assess the conservation of predicted antigenic determinants for B- (humoral immune system) and T-cells (cell-mediated immune system). In addition, predicted T-cell epitopes were docked with a pig MHC-I molecule (SLA1-04:01) in order to estimate the binding energy between the peptide and the MHC-I molecule.

## Material and methods

### Virus culture

African green monkey kidney cell line (Vero, ATCC CCL-81) was cultured in Eagle's Minimum Essential Medium (MEM) supplemented with 5% fetal bovine serum, 100 U/ml penicillin and 100 μg/ml streptomycin. The *Porcine rubulavirus* strain PAC-1 (Michoacan, Mexico, 1990) was inoculated into confluent cell cultures and incubated for 1 h at 37°C. The supernatant was discarded and the cells were washed with PBS. Fresh DMEM was added to the cells, which were then incubated for 72 h until cytopathic effects were apparent. Supernatants were clarified by centrifugation at 3,200 rpm for 30 min. Total RNA was extracted from the infected supernatants using TRIzol Reagent (Thermo Fisher Scientific) according to the manufacturer's instructions.

### Cloning and sequencing of the F gene of PRV PAC1

The open reading frame (ORF) of the F protein (PAC1) was amplified from total RNA extracted from infected supernatants by RT-PCR, using specific primers designed for this study: RuF0fw1 (5'-CCA GGA ATT CGG ATG CCA CAA CAA CAA GTT-3') and RuF0rv1 (5'-GCG GCT CTA GAA GGT ATC TAA TGA ATT TAT CTC CCA-3'). The amplicon (1633 bp) was cloned into the pJET1.2/blunt Cloning Vector (CloneJET PCR Cloning Kit, Fermentas) according to the manufacturer's instructions. The cloned product was sequenced using pJET1.2 forward and reverse sequencing primers (included in the CloneJET PCR Cloning Kit), as well as RuF1fw (5'-AAT GGA ATT CTT CAA CTA AGC CAG GCA CTT GG-3'), RuF1rv (5'-GGT AAT GTC TAG AAC AAT CTG CTC GTT CCG CA-3') and RuF2fw (5'-GCA GGA ATT CGG GGT ATC AAC ACT GA-3') primers, using the GenomeLab Dye Terminator Cycle Sequencing Kit and the automatic sequencer GenomeLab GeXP Genetic Analysis System (Beckman-Coulter, Pasadena, CA, USA). Phylogenetic analyses were conducted in MEGA7 using the Neighbor-joining method [22–24]. The analysis of the PAC1 protein sequence was performed using the SignalP-5.0 Server, the NetNGlyc 1.0 Server and the SMART Server [25–27].

### Informatic analysis of the structural proteins of PRV

The protein sequences of the RVP strains were obtained from GenBank and used for different analyzes. The sequence of the LPMV virus (1984, Accession: Y10803) was used as reference to analyze the antigenic and structural properties of the proteins under study. The physicochemical properties of the structural proteins of PRV, such as molecular weight, aliphatic index, extinction coefficient, theoretical pI, hydropathy and amino acid composition were determined using the ProtParam server (https://web.expasy.org/protparam/). The antigenicity of the proteins was determined using the VaxiJen v2.0 server (http://www.ddg-pharmfac.net/vaxijen/VaxiJen/VaxiJen.html), which makes an alignment-independent prediction based on the physicochemical properties of the proteins [28]. The target organism selected in the software was “virus”, with a default threshold of 0.4. This prediction led to the selection of antigenic proteins present in PRV for further analysis.

### Continuous and discontinuous B-cell epitope prediction

Continuous B-cell epitopes were predicted with Bepipred-1.0 Linear Epitope Prediction (http://tools.iedb.org/bcell/) [29], using a combination of a hidden Markov model and a propensity scale method at a threshold of 0.350 (sensitivity = 0.49, specificity = 0.75). The predicted epitopes were analyzed with Chou & Fasman Beta-Turn Prediction [30], Emini Surface Accessibility Prediction [31], Kolaskar and Tongaonkar Antigenicity, and Parker Hydrophilicity Prediction, using a threshold of 1.0 [32, 33].

Discontinuous B-cell epitopes of F (strain PAC1) and HN proteins (PAC1) were first modeled by homology using PHYRE2 Protein Fold Recognition Server in intensive modelling mode [34], MODELLER (https://salilab.org/modeller/). The structures were then validated using the RAMPAGE server (http://mordred.bioc.cam.ac.uk/~rapper/rampage.php). For the discontinuous epitopes, the ElliPro server (http://tools.iedb.org/ellipro/) was used [35]; this server implements Thornton's method and a residue clustering algorithm, with a minimum score of 0.5 and a maximum distance of 6 Å.

### Prediction of cytotoxic T-cell epitopes

Cytotoxic T-cell epitopes were predicted using the NetMHCpan 4.0 Server (http://www.cbs.dtu.dk/services/NetMHCpan/) through artificial neural networks [36]. The NetMHCpan 4.0 server predicts the binding of peptides to any known MHC molecule using artificial neural networks (ANNs). The method is trained on a combination of more than 180,000 quantitative binding data and mass spectroscopy derived from MHC eluted ligands. We used the swine leucocyte allele (SLA-1:0101; SLA-1:0401; SLA-1:0801), which is widely distributed in swine populations [37]. Peptide length was set to nonamers for all the selected epitopes. The threshold for a strong binder was 0.5%; for a weak binder, 2%. All peptides predicted by the NetMHCpan 4.0 Server were analyzed using ToxinPred (https://webs.iiitd.edu.in/raghava/toxinpred/design.php), a tool that predicts if a peptide is a toxin and if it can cause damage to cells [38]. For the docking simulation study, we used the crystal structure of the SLA-1:0401 molecule (PDB ID: 3QQ3) [39]. The influenza epitope, which was complexed in the binding groove of SLA-1:0401, was removed using AutoDockTools. Prior to the docking study, the nonamers predicted by the NetMHCpan 4.0 server were optimized using PEP-FOLD 3.5 [40]. The docking simulation was carried out using AutoDock Vina [41].

## Results

### Phylogenetic analysis of the F protein of PRV

**Fig 1:**
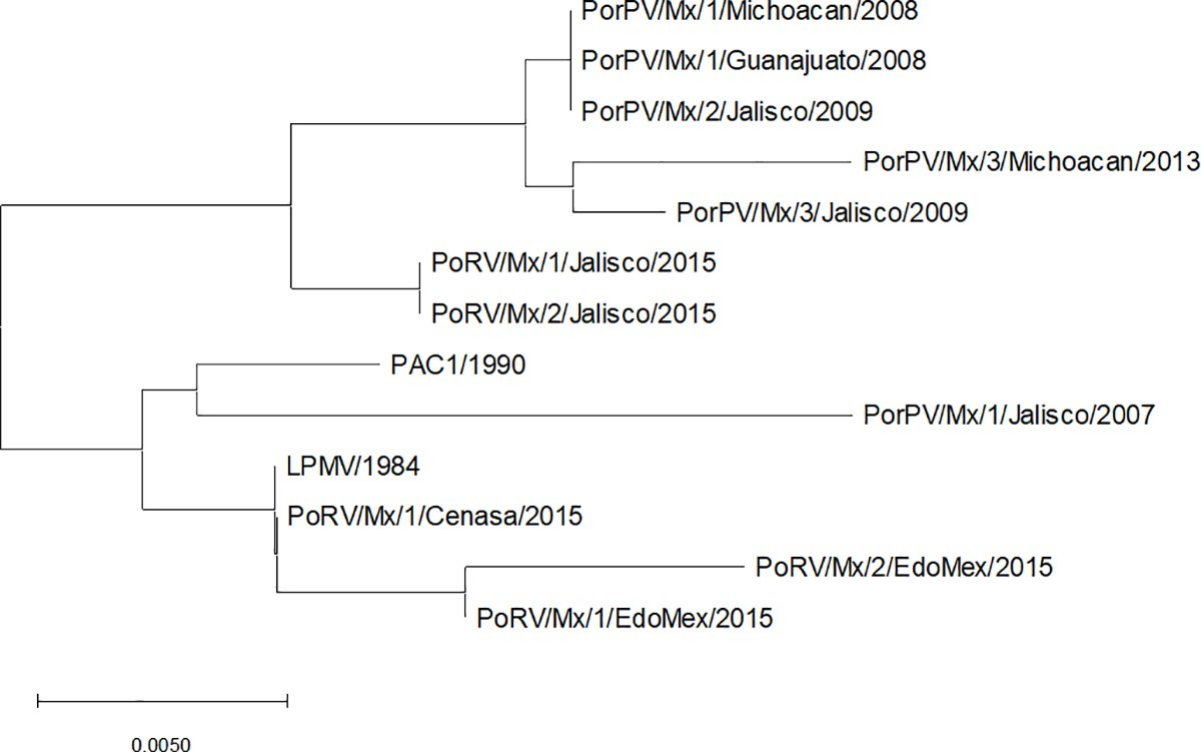
Phylogenetic tree based on the amino acid sequences of the F protein of PAC1 and other PRV strains. The evolutionary history was inferred using the Neighbor-Joining method. The figure shows the optimal tree, with the sum of the branch lengths = 0.05507775. The tree is drawn to scale, with branch lengths in the same units as those of the evolutionary distances used to infer the phylogenetic tree. The evolutionary distances are in the units of the number of amino acid substitutions per site. There were a total of 541 positions in the final dataset. Evolutionary analyses were conducted in MEGA7.

The complete coding sequence of the F protein of PAC1 (541 aa) was amplified by RT-PCR and cloned into the plasmid pJETForf. This sequence was deposited in the NCBI GenBank (MK984607) and compared to other twelve F sequences in the GenBank. The PAC1 F amino acid sequence has 97.78 to 99.26% identity with other PRV F sequences and is clustered into the group of the reference strain LPMV/1984 (Fig 1), with which it has an identity of 99.26%. Four amino acid changes were observed when compared with the reference sequence of LPMV. The F protein of the Michoacan/2013 isolate had the lowest identity with PAC1 (97.78%).

The sequence analysis of PAC1 in SignalP-5.0 found a signal peptide with a cleavage site between positions 22 and 23. The NetNGlyc 1.0 Server showed five potential N-glycosylation sites (four of them above the threshold of 0.5). The SMART server found two putative transmembrane domains at positions 106–128 and 490–512; the first one corresponded to the fusion peptide present in paramyxoviral F proteins [42].

### Immunogenic and physicochemical characterization of the structural proteins of PRV

The antigenicity of viral proteins was determined using the Vaxijen V2.0 server, selecting “virus” as target organism and a threshold of 0.4 (default) [28]. This server predicts an overall antigenicity score for each sequence (S1 Table). We used the prototype strain LPMV/1984 as a model to present the analysis results. Of all the sequences evaluated, only the P protein was not considered as an antigen (0.3560). The HN and F membrane glycoproteins were predicted as the most antigenic proteins (0.5271 and 0.5154 respectively); these sequences were subjected to further analysis.

### Prediction of continuous B-cell epitopes for PRV HN and F proteins

The epitope sequences corresponded to amino acids of the LPMV/1984 strain. Conservancy (%) indicates the fraction of protein sequences, among all PRV strains, that contained the epitope (http://tools.iedb.org/conservancy/). Continuous B-cell epitopes were predicted by the Bepipred 1.0 (IEDB server) server using a threshold of 0.350. B-cell epitopes have variable length; in the present study, we focused on linear peptides with a minimum length of 5 residues (Table 1). Eight unique linear epitopes, with 5 residues or more, were predicted for the F protein (LPMV strain), while sixteen linear epitopes were predicted for the HN protein (LPMV strain). These epitopes were evaluated using Chou & Fasman Beta-turn prediction tool, Emini Surface Accessibility, Kolaskar & Tongaonkar Antigenicity measurement tools, Parker’s Hydrophilicity index and Epitope Conservancy analysis, with a threshold of 1.0. For the F protein, three epitopes were above the threshold level of 1.0, with a conservancy of 80% (LASPDQS; PQLTNPAL and NRTYGPPAYVPPDNIIQS). For the HN protein, only one epitope was above the threshold level of 1.0, with a conservancy of 80% (PQFSQRAAASY). The conservancy results of the B-cell epitopes showed that most F epitopes were not affected by mutations presents in the F protein; in contrast, the HN epitopes were affected by these mutations, with some epitopes present in only 30.43% of the HN sequences.

**Table 1:** Continuous B-cell epitopes of the HN and F proteins of PRV (LPMV/1984).

Protein	B-cell Continuous epitopes	Position	Length	Chou (1.0)	Emini (1.0)	Kolaskar (1.0)	Parker (1.0)	Conservancy (%)
**F**	MPQQQ	1–5	5	1.012	3.215	0.987	3.18	100.00
	**LASPDQS**	**60–66**	**7**	**1.153**	**1.851**	**1.04**	**3.429**	**100.00**
	KNAEKVEQ	135–142	8	0.9	5.687	0.975	4.8	93.33
	ALGETNAA	146–153	8	0.924	0.768	0.981	2.85	100.00
	**PQLTNPAL**	**212–219**	8	**1.047**	1.499	**1.049**	**1.86**	**93.33**
	LGYGG	320–324	5	1.282	0.507	1.007	2.9	100.00
	FQEPTT	401–406	6	0.96	2.807	0.973	2.85	100.00
	**NRTYGPPAYVPPDNIIQS**	**430–447**	**18**	**1.162**	**6.479**	**1.024**	**2.144**	**100.00**
**HN**	ITSWTPD	68–74	7	1.109	1.321	0.972	1.571	91.30
	DCSSACP	116–122	7	1.269	0.335	1.12	4.286	100.00
	IGAPTES	141–147	7	1.049	0.903	0.989	3.057	100.00
	FIPTSTTTQGCT	161–172	12	1.046	0.672	1.014	2.542	95.65
	CADGGHSN	195–202	8	1.296	0.53	0.998	5.063	100.00
	IQSASDGS	210–217	8	1.177	0.995	1.001	4.412	100.00
	RSETDYYAGNSPPQ	255–268	14	1.218	15.015	0.979	4.386	82.61
	HPTGL	286–290	5	1.116	0.906	1.04	1.18	100.00
	VGSGTL	301–306	6	1.1	0.35	1.05	1.7	95.65
	**PQFSQRAAASY**	**348–358**	**11**	**1.001**	**2.581**	**1.04**	**2.409**	**100.00**
	TPPSVSSM	435–442	8	1.174	1.244	1.035	2.625	30.43
	ARPGKGGCPGNSHCP	451–465	15	1.316	0.6	1.018	3.967	34.78
	WPLTDPRSGVGGT	477–489	13	1.195	1.047	0.988	2.269	43.48
	GLDSTSERMA	496–505	10	1.038	1.655	0.954	3.46	30.43
	TQPAAYT	526–532	7	0.983	2.218	2.971	3.25	95.65
	CFRDTDTG	536–543	8	1.143	1.066	0.975	4.063	78.26

Gray shading indicates epitopes with values above 1 in all the parameters.

The epitope sequences correspond to the amino acids of the LPMV/1984 strain. Conservancy (%) is defined as the fraction of protein sequences, among all PRV strains, that contained the epitope (http://tools.iedb.org/conservancy/).

### Prediction of discontinuous B-cell epitopes for the HN and F proteins of PRV

The epitope sequences correspond to the amino acids of the LPMV/1984 strain. The ElliPro score of each epitope is defined as a protrusion index value averaged over epitope residues; values ≥0.5 are considered significant for a continuous epitope [35]. The structure of the HN and F proteins was predicted by homology with MODELLER and PHYRE2, using *ab initio* structure prediction algorithms for the transmembrane domains. The two models were validated using the RAMPAGE server. In the HN proteins, 93.9% of the residues were in favored regions, 4.5% residues in allowed regions and 1.6% in outlier regions. In the F proteins, 93.1% of the residues were in favored regions, 5.9% in allowed regions and 0.9% in outlier regions.

**Fig 2:**
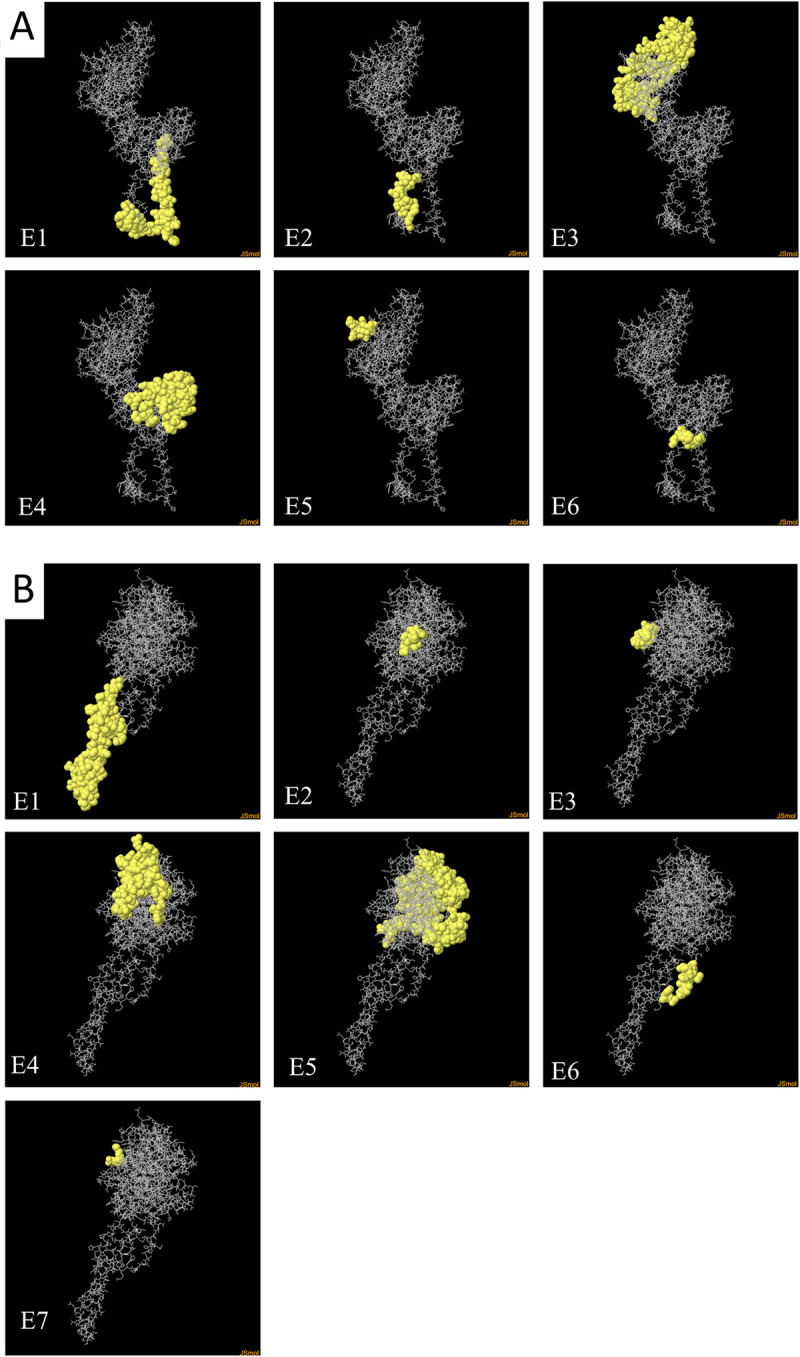
Discontinuous B-cell epitopes for the F and HN proteins predicted by the ElliPro server. The predicted epitope residues are shown as yellow balls. A) Discontinuous epitopes of the F protein 1–6 (E1-E6). B) Discontinuous epitopes of the HN protein 1–7 (E1-E7). Visualization done using Jmol software.

Discontinuous B-cell epitopes were predicted using the ElliPro server. Six epitopes were predicted for the F protein (S2 Table); the epitopes with the highest score (0.872) were located in the stalk of the F protein (Fig 2). Seven epitopes were predicted for the HN protein, four of them localized in the head domain of the protein.

### Prediction of cytotoxic T-cell epitopes for the HN and F proteins of PRV

**Fig 3:**
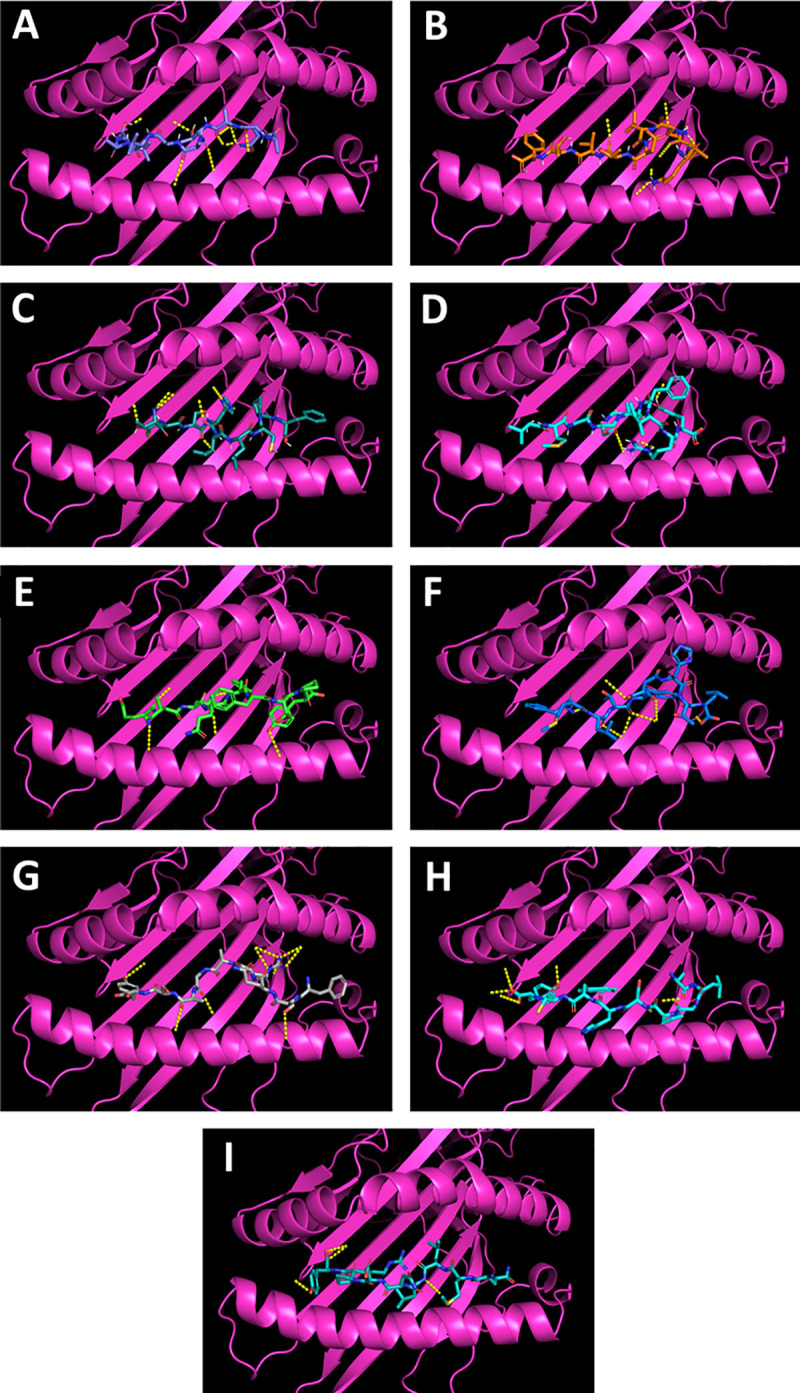
Binding patterns of F and HN epitopes for SLA-01:0401. The predicted epitope residues are shown as lines. The SLA-01:0401 is shown in magenta. F epitopes: A) AQATAAVAL, B) KVQLDTLTF, C) TMSHILCPF, D) VMGDKFIRY. HN epitopes: E) EINQFFTPY, F) FMLTFDHTL, G) FSQRAAASY, H) ALGPSHWCY and I) QMLLNDPRY. The yellow dash lines indicate hydrogen bonds between the peptide and the SLA molecule. Visualization done using PyMol software.

For cytotoxic T-cell epitopes, we used the NetMHCpan 4.0 Server (DTU Bioinformatics), which predicts the binding of MHC-I peptides using artificial neural networks. The MHCI alleles used for the analysis were SLA-1*01:01, SLA-1*04:01 and SLA-1*08:01. These alleles were widely distributed in the pig population [37, 43]. The S3 Table shows the peptides that were strong binders to the selected MHCI molecules (the threshold for a strong binder was 0.5%; for a weak binder, 2%). Twenty-nine cytotoxic epitopes were obtained for the F protein and 34 for the HN protein. The putative immunogenicity of the peptides was assessed using the Class I Immunogenicity server (IEDB). Sixteen peptides predicted for the F protein and twenty-two peptides predicted for the HN protein were predicted to be immunogenic. ToxinPred is an *in silico* method that predicts whether a peptide is a toxin that can cause damage to cells. ToxinPred uses a support vector machine (SVM) to predict toxicity along with mutations [38]. All peptides were predicted to be non-toxic. We selected the peptides that were recognized by two alleles; they had positive immunogenicity and a conservancy percentage of 100% (S3 Table). The highest number of peptides was found in the F1 peptide fragment, which may thus be proposed as the immunogenic region of the protein. In the docking simulation, the box center coordinates of the binding groove of SLA-1:0401 were X = 18.213, Y = 2.001 and Z = 41.238, and the grid box size was X = 30, Y = 30 and Z = 30. Table 2 shows the binding energy values of the predicted epitopes for the receptor of SLA-01:0401. These values suggest that all the F protein epitopes fit into the binding groove of the SLA molecule (Fig 3). AQATAAVAL has a binding energy of -7.0 kcal/mol. The five HN epitopes also fit into the binding groove. FSQRAAASY has a binding energy of -7.8 kcal/mol.

**Table 2:** Binding energy of the predicted epitopes for SLA-01*04:01 allele using Autodock Vina.

Protein	Peptide sequence	Binding energy (kcal/mol)
F protein	AQATAAVAL	-7.0
	TMSHILCPF	-5.8
	KVQLDTLTF	-6.6
	VMGDKFIRY	-6.3
HN protein	FMLTFDHTL	-7.3
	QMLLNDPRY	-6.4
	ALGPSHWCY	-7.3
	EINQFFTPY	-6.8
	FSQRAAASY	-7.8

## Discussion

Many emerging diseases have appeared in recent years. Zoonosis can be a major problem, causing previously unreported infections in humans. This is why it is so important to develop vaccines against viral diseases affecting in animals in close contact with humans. It has already been seen that pigs can transmit diseases such as Swine Influenza; thus, attention should be paid to other viruses that affect pigs such as PRV. In fact, it has been speculated that PRV may originate from bats, like other paramyxoviruses that infect animals and humans [5].

Due to the persistence of BED outbreaks in Mexico and the lack of data on the effectiveness of existing vaccines, PRV infection is a major problem for pig farms in Mexico. For these reasons, we need to understand the behavior of antigenic determinants in the structural proteins of the virus and how mutations affecting these determinants could serve to develop effective prevention strategies. The F protein is a surface protein with a high percentage of identity among all reported PRV strains (97.78 to 99.26%). We propose to consider the F protein as a possible antigen that could provide protection against different strains of PRV. None of the substitutions in the F protein cleavage site (HRKKR) have been found. Some reports suggest that the cleavage site in the F protein participates in the virulence of PRV [5, 9]. There are been reports that the surface PRV proteins, HN and F, are the most antigenic components of other paramyxoviruses (e.g. MuV, Measles virus and PIV5) [18, 20, 44].

Currently, several many approaches are being considered to design possible vaccines for PRV. The advantages of immunoinformatic tools have already been demonstrated for other pathogens such as Ebola virus, Zika virus or the Oropouche virus [45–47]. The antigenic capacity of the structural proteins of the PRV reference strain LPMV was evaluated using the Vaxijen V2.0 server. F, HN and NP were predicted as the most antigenic structural proteins, with a score > 0.5. Other studies have used the Vaxijen server to select promising antigens against the Human Immunodeficiency Virus (HIV) and the Hepatitis C virus, structural proteins with a Vaxijen score > 0.4 [48, 49].

Antigenic determinants recognized by B-cells are important because they can induce the immune system of an organism to elicit memory protection mediated by producing antibodies with the ability to act quickly against reinfection by PRV. In some paramyxoviruses, the presence of these antibodies is enough to neutralize the infection. There are reports of antibodies with neutralizing activity that can recognize the HN and F proteins of MuV, Newcastle disease virus, Measles virus or Nipah virus (F protein) [19, 50, 51]. The memory response of antibodies in surviving pigs, in the case of a PRV infection, suggests that it is long-lasting; that is why determining possible antigenic regions that can activate the response of B-cells is so important [52]. The results of the present study predict that the HN protein contains more antigenic determinants for B-cells (16 epitopes) than the F protein (8 epitopes). BepiPred is a reliable tool that has already been used in immunoinformatics for the possible design of vaccines against emerging viruses such as SARS-CoV-2 [53]. For HN, only PQFSQRAAASY, and for the F protein, only LASPDQS, PQLTNPAL and NRTYGPPAYVPPDNIIQS met the criteria, based on the evaluated parameters, that is, Kolaskar and Tongaonkar antigenicity, the Chou & Fasman method and Parker’s Hydrophilicity, which have been used not only to predict which region of a protein is antigenic, but even to determine how some mutations can affect the antigenicity of an epitope [54]. The ATRSETDYY and AAYTTTTCF epitopes of the HN protein are peptides with an important immunogenic capacity, since they are recognized as antigenic determinants for B cells. Furthermore, it has already been reported *in vitro* that these epitopes are recognized by antibodies generated during PRV infection. Zenteno et al. (2007) studied some peptides that have common sequences with two of the epitopes that we propose in present study (ATRSETDYY and AAYTTTTCF). Those peptides were able to induce antibodies in mice and one of them was able to inhibit the hemagglutinating activity of PRV, which suggests that these peptides may possibly be involved in the recognition of carbohydrates that are part of the receptor for the virus [55]. These findings suggest that it is possible that antibodies directed against these epitopes could neutralize the infection.

In the selection of epitopes, priority was given to those with high conservation among the PRV strains. The antigenic determinants in the F protein are preserved compared to other known PRV sequences, which suggests that the F protein epitopes used as antigens could be very useful targets against different PRV strains. The relative low conservation (30.43% was the lowest value) of some antigenic determinants of the HN protein (Table 1) may be related to the antigenic diversity reported in other studies on PRV [12, 13]. As far as we know, this is the first report on how mutations in the HN protein sequence can directly affect the predicted antigenic determinants. Although there are few mutations in the HN protein [5], they affect the region comprising residues 435–509. The globular region of the HN protein contains most of the continuous and discontinuous antigenic determinants.

MHC-I antigenic determinants activate an immune cellular response. This type of response normally activates cytotoxic cells that lyse the cells infected by PRV. The occurrence of this response can be determined through the presence of HLA alleles in the host organism. One way to predict the behavior of these antigenic determinants is to evaluate their binding capacity to MHCI and to evaluate their immunogenic capacity by molecular docking [47, 56]. In pigs, the major histocompatibility complex (MHC in pigs) and the swine leukocyte antigen (SLA) have an important role mediating cellular immunity, which can eliminate viruses and recognize other antigens in pigs. However, this allele is highly polymorphic, which can cause difficulties for the detection of epitopes suitable for a vaccine, That is why the SLA-1 04:01 and SLA-1 08:01 alleles were used for the analysis of antigenic determinants, which have been reported as alleles with a wide distribution in pigs [57, 58]. The NetMHCpan 4.0 and Class I Immunogenicity server are widely used tools for the design of vaccines with the possibility of activating the host cellular response mediated by MHC I. There are few reports of the use of this tools to predict epitopes against Hepatitis C virus or Herpes simplex virus vaccines [59, 60]. These tools predicted sixteen peptides for the F protein and twenty-two peptides for the HN protein to be immunogenic. We only analyzed 100% conservancy epitopes (4 and 5 epitopes for F protein and HN protein respectively). The binding energies for these epitopes ranged from -5.8 to -7.8 kcal/mol. The binding energies of all predicted peptides were within a range similar to those reported in a docking analysis with MHC-I molecules for other viruses, including the herpes simplex virus or the Saint Louis encephalitis virus [61, 62].

An important result of the present work is that none of the predicted peptides are toxic, which means that any epitope predicted could be used. Prediction of peptide toxicity is based on the recognition of motifs that are present in proteins or peptides that are experimentally known to be toxic. In similar studies, some peptides with immunogenic capacity have been shown to be toxic and must be discarded [63], but this feature apparently is not present in the selected HN and F peptides [14, 55].

The NRTYGPPAYVPPDNIIQS peptide in protein F may also be important because, in addition to being considered an antigenic determinant for B cells and MHCI T cells, it has the theoretical capacity of inducing an immune response.

This type of study provides an overview of the possible elements that could be used to generate a PRV vaccine. One of the possible strategies is to generate a chimeric protein that contains the epitopes of the HN and F proteins in a single antigen. There are examples of chimeric proteins that are intended to be preventive treatments against HIV and the Influenza virus [64, 65]. Zenteno et al. reported that it is possible to use peptides to induce a response that could affect proteins that participate in the infectious process of PRV. They also mentioned that, besides vaccines, immunogenic peptides can be used as rapid diagnostic tools for PRV [55]. Another approach is to generate recombinant HN and F proteins, either by expressing all the protein or only the regions that concentrate the greatest amount of B and T cell antigenic determinants, and thereby generate a divalent vaccine. In a previous work, our group showed that a recombinant HN protein (PAC1 strain) was capable of inducing the production of neutralizing antibodies in a murine model [14]. Some examples of this kind of vaccines are those used against the Human papillomavirus or the Ebola virus [66, 67].

## Conclusion

The need to prevent blue eye disease in pigs led us to study proteins or peptides with immunogenic capacity against PRV. Bioinformatics analysis allows to estimate the behavior of a protein in vivo. In the present study, we sequenced the F protein of the PAC1 strain, phylogenetically analyzed the F protein of PRV and predicted potential antigenic determinants of the HN and F proteins of PRV. Antigenic determinants recognized by B cells and T cells, as well as the structure of the F and HN proteins, were determined by homology modelling, which allowed us to predict conformational epitopes. This study could lead to the use of these two proteins as antigens, obtaining recombinant proteins, or only peptides, that could activate different responses in the immune system, which in turn could help optimize time and costs in the development of new vaccines or diagnostic tools.

## Supporting information

S1 Table Prediction of the immunogenic and physicochemical properties of the structural proteins of PRV (LPMV/1984 strain). (DOCX)

S2 Table Discontinuous B-cell epitopes of HN and F proteins (LPMV/1984). (DOCX)

S3 Table List of Predicted strong binding cytotoxic T-cell epitopes of HN and F proteins (LPMV/1984). (DOCX)
