# Supplementary material for: Immunoinformatics approach for predicting epitopes in HN and F proteins of Porcine rubulavirus
Source: PLoS One. 2020 Sep 25;15(9):e0239785. doi: 10.1371/journal.pone.0239785 (PMC7518572; doi:10.1371/journal.pone.0239785)
Supplement: S1 Table — (DOCX) [file pone.0239785.s001.docx]

**S1 Table. Prediction of the immunogenic and physicochemical properties of the structural proteins of PRV (LPMV/1984 strain)**.

| **Protein** | **Length (aa)** | **Molecular Weight**  **(Da)** | **Isoelectric point** | **Extinction Coefficient (M^−1^ cm−1)** | **-R (Asp + Glu)** | **+ R (Arg + Lys)** | **Aliphatic Index** | **GRAVY^a^** | **Vaxijen score^b^** |
| --- | --- | --- | --- | --- | --- | --- | --- | --- | --- |
| F | 541 | 58311.66 | 6.15 | 43610 | 35 | 31 | 119.52 | 0.371 | 0.5084 |
| HN | 576 | 63324.82 | 8.44 | 88335 | 30 | 36 | 85.38 | 0.089 | 0.5271 |
| M | 369 | 41657.72 | 9.45 | 37275 | 33 | 48 | 89.08 | -0.183 | 0.4645 |
| NP | 545 | 60096.80 | 5.30 | 50560 | 63 | 53 | 88.22 | -0.191 | 0.5101 |
| L | 2251 | 255269.74 | 6.33 | 307795 | 245 | 225 | 97.64 | -0.102 | 0.4163 |
| P | 404 | 42475.98 | 5.49 | 17085 | 43 | 38 | 77.33 | -0.372 | 0.3560 |

Predicted values were calculated considering the amino acid content of the proteins. The analysis was performed using the Vaxijen V2.0 server.

^a^GRAVY (grand average of hydropathy).

^b^Denotes a probable antigenic protein (Vaxijen score ≥0.4).
