# Supplementary material for: Immunoinformatics approach for predicting epitopes in HN and F proteins of Porcine rubulavirus
Source: PLoS One. 2020 Sep 25;15(9):e0239785. doi: 10.1371/journal.pone.0239785 (PMC7518572; doi:10.1371/journal.pone.0239785)
Supplement: S2 Table — (DOCX) [file pone.0239785.s002.docx]

**S2 Table. Discontinuous B-cell epitopes of HN and F proteins (LPMV/1984).**

| **Protein** | **No.** | **Residues** | **Number of residues** | **Score** |
| --- | --- | --- | --- | --- |
| **F protein** | 1 | _:E448, _:L450, _:S453, _:G454, _:N455, _:L456, _:I457, _:A458, _:V459, _:N460, _:N461, _:S462, _:L463, _:S464, _:S465, _:A466, _:L467, _:N468, _:H469, _:L470, _:A471, _:T472, _:S473, _:E474, _:I475, _:L476, _:R477, _:K478, _:Q479, _:Q480, _:I481, _:W482, _:T483, _:S484, _:S485, _:L486, _:G487, _:I488, _:S489, _:T490, _:I491, _:V492, _:A493, _:L494, _:V495, _:I496, _:I497, _:G498, _:I499, _:L500, _:I501, _:I502, _:C503, _:L504, _:V505, _:V506, _:T507, _:W508, _:A509, _:A510, _:L511, _:W512, _:A513, _:L514, _:L515, _:K516, _:E517 | 67 | 0.872 |
|  | 2 | _:V518, _:R519, _:G520, _:L521, _:N522, _:S523, _:A524, _:V525, _:N526, _:S527, _:Q528, _:L529, _:S530, _:S531, _:Y532, _:V533 | 16 | 0.763 |
|  | 3 | _:P58, _:S59, _:L60, _:A61, _:S62, _:P63, _:D64, _:Q65, _:S66, _:C67, _:A68, _:L69, _:H70, _:S71, _:I72, _:I73, _:N74, _:N76, _:A77, _:T78, _:Q80, _:A81, _:S84, _:A87, _:E88, _:N91, _:L92, _:I93, _:S94, _:T95, _:A96, _:L97, _:R98, _:E99, _:Q100, _:H101, _:R102, _:K103, _:K104, _:R105, _:F106, _:A107, _:G108, _:V109, _:A110, _:I111, _:G112, _:L113, _:T114, _:A115, _:L116, _:G117, _:V118, _:A119, _:V131, _:N134, _:K135, _:E138, _:K139, _:E141, _:Q142, _:L143, _:S144, _:Q145, _:A146, _:G148, _:E149, _:T150, _:N151, _:A152, _:Q169, _:I171, _:Q172, _:N173, _:Q174, _:I175, _:N176, _:T177, _:A178, _:I179, _:L180, _:P181, _:Q182, _:I183, _:H184, _:N185, _:L186, _:S187, _:C188, _:Q189, _:V190, _:I191, _:D192, _:A193, _:Q194, _:L195, _:G196, _:N197, _:I198, _:L199, _:S200, _:N216, _:N266, _:L267, _:Q268, _:N269, _:M270 | 107 | 0.696 |
|  | 4 | _:M1, _:P2, _:Q3, _:I23, _:V364, _:V365, _:G366, _:S367, _:F368, _:L369, _:R371, _:N376, _:G377, _:V378, _:V379, _:N382, _:C383, _:A384, _:D385, _:M386, _:S387, _:C388, _:V389, _:C390, _:F391, _:D392, _:P393, _:Q394, _:E395, _:I396, _:I397, _:Y398, _:Q399, _:N400, _:F401, _:Q402, _:E403, _:V407, _:I408, _:D409, _:I410, _:K411, _:K412, _:C413, _:G414, _:K415, _:V416, _:Q417, _:L418, _:D419, _:T420, _:L421, _:T422, _:F423, _:T424, _:I425, _:S426, _:T427, _:F428, _:A429, _:N430, _:R431, _:T432, _:Y433, _:G434 | 65 | 0.68 |
|  | 5 | _:D156, _:L157, _:I158, _:D159, _:A160, _:T161, _:K162, _:N163, _:L164 | 9 | 0.665 |
|  | 6 | _:D536, _:K537, _:F538, _:I539, _:R540, _:Y541 | 6 | 0.587 |
| **HN protein** | 1 | _:R23, _:L24, _:C25, _:F26, _:R27, _:I28, _:F29, _:L31, _:H49, _:T50, _:L51, _:L52, _:T53, _:T54, _:T55, _:Q56, _:F57, _:L58, _:T59, _:S60, _:I61, _:G62, _:N63, _:L64, _:Q65, _:S66, _:T67, _:I68, _:T69, _:S70, _:W71, _:T72, _:P73, _:D74, _:V75, _:Q76, _:A77, _:M78, _:L79, _:S80, _:I81, _:S82, _:N83, _:Q84, _:L85, _:I86, _:Y87, _:T88, _:T89, _:S90, _:I91, _:T92, _:L93, _:P94, _:L95, _:K96, _:I97, _:S98, _:T99, _:T100, _:E101, _:M102, _:S103, _:I104, _:L105, _:T106, _:A107, _:I108, _:R109, _:D110, _:H111, _:C112, _:H113, _:C114, _:P115, _:D116, _:S118, _:S119, _:C121 | 79 | 0.865 |
|  | 2 | _:Q211, _:S212, _:A213, _:S214, _:D215, _:G216, _:S217, _:P218, _:L219 | 9 | 0.731 |
|  | 3 | _:N431, _:F432, _:L433, _:T434, _:T435, _:P436, _:P437, _:S438, _:V439 | 9 | 0.726 |
|  | 4 | _:N129, _:D130, _:P131, _:R132, _:Y133, _:M134, _:S135, _:G136, _:F140, _:I141, _:G142, _:A143, _:P144, _:T145, _:E146, _:S147, _:I148, _:N149, _:I150, _:T151, _:F152, _:G153, _:P154, _:L155, _:F156, _:G157, _:I158, _:P159, _:S160, _:P181, _:L479, _:T480, _:D481, _:P482, _:S484, _:G485, _:V486, _:G487, _:G488, _:T489, _:S490, _:L491, _:R538, _:D539, _:T540, _:D541, _:T542, _:G543, _:K544, _:I545, _:Y546, _:R568, _:E569, _:I570, _:K571, _:I572, _:Q573, _:S574, _:R575, _:Y576 | 60 | 0.676 |
|  | 5 | _:T164, _:S165, _:T166, _:T167, _:T168, _:Q169, _:G170, _:T192, _:A193, _:G194, _:C195, _:A196, _:D197, _:G198, _:G199, _:H200, _:S201, _:N233, _:A253, _:T254, _:R255, _:S256, _:T258, _:D259, _:Y260, _:Y261, _:A262, _:G263, _:N264, _:S265, _:P266, _:P267, _:P287, _:T288, _:G289, _:L290, _:A291, _:N292, _:G293, _:V295, _:G318, _:M319, _:Q320, _:Q321, _:I322, _:L323, _:A324, _:N325, _:Q326, _:S327, _:G328, _:E329, _:I330, _:N331, _:Q332, _:F333, _:F334, _:T335, _:P336, _:N338, _:A339, _:T340, _:V341, _:R342, _:C343, _:A344, _:M345, _:A346, _:Q347, _:P348, _:Q349, _:F350, _:S351, _:Q352, _:R353, _:A354, _:A355, _:A356, _:S357, _:Y358, _:Y359, _:P360, _:R361, _:F363, _:S364, _:N365, _:R366, _:W367, _:Y381, _:Q382, _:T383, _:Q384, _:L390, _:P391, _:N392, _:R393, _:M394, _:V395, _:M397, _:W419, _:E448, _:P453, _:G454, _:K455, _:G456, _:G457, _:P459, _:G460, _:N461, _:S462, _:H463, _:C464, _:P465, _:A466, _:T467, _:C468, _:T470, _:D498, _:S499, _:T500, _:S501, _:E502, _:M504, _:A505, _:I512, _:G513, _:E514, _:S515, _:L516, _:L517, _:S518, _:K519, _:T520, _:Y521, _:L522, _:L523, _:S524, _:K525, _:T526, _:Q527, _:P528, _:A529, _:G554, _:K555, _:V556, _:L557, _:L558, _:G559, _:E560 | 149 | 0.626 |
|  | 6 | _:I9, _:M10, _:H11, _:L12, _:A13, _:Q14, _:P15, _:A16, _:I17, _:A18, _:T21, _:W22 | 12 | 0.569 |
|  | 7 | _:G407, _:D408, _:R409 | 3 | 0.502 |

The epitope sequences correspond to the amino acids of the LPMV/1984 strain. The score of each epitope is defined as a protrusion index value averaged over epitope residues; values ≥0.5 are considered significant for a continuous epitope.
