# Supplementary material for: Immunoinformatics approach for predicting epitopes in HN and F proteins of Porcine rubulavirus
Source: PLoS One. 2020 Sep 25;15(9):e0239785. doi: 10.1371/journal.pone.0239785 (PMC7518572; doi:10.1371/journal.pone.0239785)
Supplement: S3 Table — (DOCX) [file pone.0239785.s003.docx]

**S3 Table. List of Predicted strong binding cytotoxic T-cell epitopes of HN and F proteins (LPMV/1984).**

| **Protein** | **Peptide sequence** | **Position** | **Allele** | **Affinity** | **%rank** | **Immunogenicity** | **Toxicity** | **Conservancy** |
| --- | --- | --- | --- | --- | --- | --- | --- | --- |
| **F protein** | VTNVRQLAY | 35 | SLA-1:0401; SLA-1:0801 | 225.2; 493 | 0.1733; 0.3729 | -0.38559 | Non-toxin | **6.67%** |
|  | YTQAGSTYL | 44 | SLA-1:0101; SLA-1:0401; SLA-1:0801 | 880.3;352.6; 566.1 | 0.0234; 0.2668; 0.4247 | -0.09036 | Non-toxin | **100.00%** |
|  | SIINYNATL | 71 | SLA-1:0101 | 4084.9 | 0.4736 | 0.0827 | Non-toxin | **93.33%** |
|  | ILSPIAENL | 82 | SLA-1:0101 | 3731.1 | 0.397 | 0.18229 | Non-toxin | **100.00%** |
|  | TAAQATAAV | 120 | SLA-1:0101 | 2424.2 | 0.1769 | 0.02666 | Non-toxin | **100.00%** |
|  | **AQATAAVAL** | **122** | **SLA-1:0101; SLA-1:0801** | **3079.5; 539.6** | **0.2805; 0.405** | **0.18439** | **Non-toxin** | **100.00%** |
|  | KVEQLSQAL | 139 | SLA-1:0101 | 3419.8 | 0.3423 | -0.32549 | Non-toxin | **93.33%** |
|  | AQLGNILSL | 193 | SLA-1:0801 | 345.8 | 0.2643 | 0.04346 | Non-toxin | **93.33%** |
|  | QLGNILSLY | 194 | SLA-1:0401; SLA-1:0801 | 404; 146.4 | 0.306; 0.0974 | -0.02245 | Non-toxin | **93.33%** |
|  | YLTELTTVF | 202 | SLA-1:0401; SLA-1:0801 | 651.1; 117.9 | 0.4918; 0.0699 | 0.19597 | Non-toxin | **93.33%** |
|  | LTNPALSPL | 214 | SLA-1:0101 | 1756.4 | 0.1007 | -0.1317 | Non-toxin | **93.33%** |
|  | LGDLMSSGL | 249 | SLA-1:0101 | 3256.8 | 0.3116 | -0.45051 | Non-toxin | **100.00%** |
|  | STAKVLDLV | 285 | SLA-1:0101 | 2259.8 | 0.1547 | -0.1623 | Non-toxin | **100.00%** |
|  | VLELGSEVL | 312 | SLA-1:0101 | 3419.4 | 0.3422 | 0.00723 | Non-toxin | **100.00%** |
|  | ELGSEVLGY | 314 | SLA-1:0801 | 522.6 | 0.3931 | -0.00867 | Non-toxin | **100.00%** |
|  | **TMSHILCPF** | **330** | **SLA-1:0401; SLA-1:0801** | **584.6; 149.9** | **0.4437; 0.1001** | **0.04603** | **Non-toxin** | **100.00%** |
|  | RVLSTDMKY | 342 | SLA-1:0401; SLA-1:0801 | 239.2; 220.5 | 0.1836; 0.1554 | -0.00588 | Non-toxin | **100.00%** |
|  | FSPVVGSFL | 361 | SLA-1:0101 | 2931.5 | 0.2551 | 0.03882 | Non-toxin | **100.00%** |
|  | FALVNGVVI | 372 | SLA-1:0101 | 4050.1 | 0.4662 | 0.1225 | Non-toxin | **100.00%** |
|  | CADMSCVCF | 383 | SLA-1:0101 | 3773.9 | 0.4057 | -0.37801 | Non-toxin | **100.00%** |
|  | CFDPQEIIY | 390 | SLA-1:0401 | 468.9 | 0.3575 | 0.16757 | Non-toxin | **100.00%** |
|  | **KVQLDTLTF** | **415** | **SLA-1:0401; SLA-1:0801** | **179.6;303.3** | **0.1332; 0.2285** | **0.0227** | **Non-toxin** | **100.00%** |
|  | QLDTLTFTI | 417 | SLA-1:0101 | 1194.3 | 0.0499 | 0.19348 | Non-toxin | **100.00%** |
|  | TLTFTISTF | 420 | SLA-1:0801 | 412.5 | 0.3109 | 0.17654 | Non-toxin | **100.00%** |
|  | RTYGPPAYV | 431 | SLA-1:0101 | 4108.4 | 0.4785 | 0.04252 | Non-toxin | **100.00%** |
|  | SLSSALNHL | 462 | SLA-1:0101 | 3295 | 0.3189 | -0.17907 | Non-toxin | **100.00%** |
|  | TSSLGISTI | 483 | SLA-1:0101 | 3976.5 | 0.4505 | -0.02352 | Non-toxin | **100.00%** |
|  | AVNSQLSSY | 524 | SLA-1:0401; SLA-1:0801 | 39.1; 77 | 0.0195; 0.032 | -0.52809 | Non-toxin | **100.00%** |
|  | **VMGDKFIRY** | **533** | **SLA-1:0401; SLA-1:0801** | **556; 237.3** | **0.4212; 0.17** | **0.07608** | **Non-toxin** | **100.00%** |
| **HN protein** | MSQLGTDQI | 1 | SLA-1:0101 | 2346.7 | 0.1666 | -0.02818 | Non-toxin | **100.00%** |
|  | **FMLTFDHTL** | **43** | **SLA-1:0101** | **2038** | **0.1276** | **0.22032** | **Non-toxin** | **100.00%** |
|  | AMLSISNQL | 77 | SLA-1:0801 | 530 | 0.398 | -0.26934 | Non-toxin | **100.00%** |
|  | YTTSITLPL | 87 | SLA-1:0101; SLA-1:0801 | 873.6; 447.8 | 0.0231; 0.3394 | -0.00357 | Non-toxin | **100.00%** |
|  | **QMLLNDPRY** | **125** | **SLA-1:0401; SLA-1:0801** | **575.6; 350.3** | **0.4367; 0.2675** | **0.0207** | **Non-toxin** | **100.00%** |
|  | MLLNDPRYM | 126 | SLA-1:0801 | 362.7 | 0.2761 | 0.04257 | Non-toxin | **100.00%** |
|  | **ALGPSHWCY** | **178** | **SLA-1:0401; SLA-1:0801** | **198.1; 170.6** | **0.1506; 0.1145** | **0.02463** | **Non-toxin** | **100.00%** |
|  | QSASDGSPL | 211 | SLA-1:0101 | 2854.9 | 0.2425 | -0.24637 | Non-toxin | **100.00%** |
|  | SASDGSPLL | 212 | SLA-1:0101 | 1374.2 | 0.0606 | -0.16995 | Non-toxin | **100.00%** |
|  | ASDGSPLLI | 213 | SLA-1:0101; SLA-1:0401 | 600.2; 477.7 | 0.0147; 0.364 | -0.14608 | Non-toxin | **95.65%** |
|  | LLITARSYY | 219 | SLA-1:0801 | 148.9 | 0.0993 | 0.0273 | Non-toxin | **82.61%** |
|  | AVVPGGCAM | 240 | SLA-1:0801 | 292 | 0.2173 | 0.0445 | Non-toxin | **100.00%** |
|  | ATRSETDYY | 253 | SLA-1:0401 | 348.3 | 0.2634 | 0.00093 | Non-toxin | **95.65%** |
|  | TLYNEYLLF | 305 | SLA-1:0401; SLA-1:0801 | 608.1; 106.4 | 0.4613; 0.0585 | 0.07047 | Non-toxin | **95.65%** |
|  | **EINQFFTPY** | **329** | **SLA-1:0401; SLA-1:0801** | **271.8; 236.8** | **0.2068; 0.1696** | **0.13182** | **Non-toxin** | **100.00%** |
|  | **FSQRAAASY** | **350** | **SLA-1:0401; SLA-1:0801** | **260.2; 566.9** | **0.198; 0.4253** | **0.02577** | **Non-toxin** | **100.00%** |
|  | SQRAAASYY | 351 | SLA-1:0801 | 219.5 | 0.1545 | -0.01068 | Non-toxin | **100.00%** |
|  | AAASYYPRY | 354 | SLA-1:0401; SLA-1:0801 | 278.2; 236.8 | 0.2119; 0.1696 | -0.13997 | Non-toxin | **100.00%** |
|  | RSAIVACPY | 369 | SLA-1:0401; SLA-1:0801 | 540.9; 617.7 | 0.409; 0.4605 | 0.17167 | Non-toxin | **95.65%** |
|  | QTQCTLIPL | 382 | SLA-1:0101 | 2022.2 | 0.1256 | 0.04135 | Non-toxin | **65.22%** |
|  | MMGSEGRIF | 396 | SLA-1:0801 | 445.8 | 0.3379 | 0.09537 | Non-toxin | **100.00%** |
|  | FTLGDRLFY | 404 | SLA-1:0401; SLA-1:0801 | 422.2; 319.6 | 0.3213; 0.2439 | 0.15986 | Non-toxin | **95.65%** |
|  | TLGDRLFYY | 405 | SLA-1:0401; SLA-1:0801 | 334.3; 177.3 | 0.2519; 0.1189 | 0.16992 | Non-toxin | **95.65%** |
|  | SSSWWPYPL | 416 | SLA-1:0101 | 484.4 | 0.01 | 0.36485 | Non-toxin | **100.00%** |
|  | SSWWPYPLL | 417 | SLA-1:0101 | 1620 | 0.0851 | 0.26467 | Non-toxin | **100.00%** |
|  | SWWPYPLLY | 418 | SLA-1:0801 | 674.2 | 0.4964 | 0.03086 | Non-toxin | **100.00%** |
|  | LLYQVGLNF | 424 | SLA-1:0801 | 312.7 | 0.2375 | -0.0588 | Non-toxin | **95.65%** |
|  | VSSMTQVPL | 439 | SLA-1:0101 | 1661.5 | 0.0894 | -0.27328 | Non-toxin | **95.65%** |
|  | MTQVPLEHL | 442 | SLA-1:0101 | 3435 | 0.345 | 0.0861 | Non-toxin | **91.30%** |
|  | GVYADVWPL | 471 | SLA-1:0801 | 657.9 | 0.4864 | 0.27909 | Non-toxin | **95.65%** |
|  | GLDSTSERM | 496 | SLA-1:0101; SLA-1:0401 | 3605.9; 160.6 | 0.3756; 0.1184 | -0.16246 | Non-toxin | **30.43%** |
|  | STSERMAPV | 499 | SLA-1:0101 | 4076.9 | 0.4719 | -0.04131 | Non-toxin | **26.09%** |
|  | MAPVNYLAI | 504 | SLA-1:0101 | 2548.8 | 0.1936 | 0.04166 | Non-toxin | **73.91%** |
|  | AAYTTTTCF | 529 | SLA-1:0801 | 378.2 | 0.2863 | 0.11346 | Non-toxin | **100.00%** |

Gray shading: antigenic determinants that are recognized by two or more alleles and have a conservancy of 100 %. Conservancy (%) is defined as the fraction of protein sequences, among all PRV strains, that contain the epitope (http://tools.iedb.org/conservancy/).
